# Complications and management of acute copper sulphate poisoning; a case discussion

**DOI:** 10.1186/1745-6673-6-34

**Published:** 2011-12-19

**Authors:** Champika SSK Gamakaranage, Chaturaka Rodrigo, Sajitha Weerasinghe, Ariaranee Gnanathasan, Visvalingam Puvanaraj, Harshani Fernando

**Affiliations:** 1 University Medical Unit, National Hospital of Sri Lanka, Colombo, Sri Lanka; 2 Department of Clinical Medicine, Faculty of Medicine, University of Colombo, Sri Lanka; 3 Ward 49, National Hospital of Sri Lanka, Colombo, Sri Lanka; 4 Medical Intensive Care Unit, National Hospital of Sri Lanka, Colombo, Sri Lanka

## Abstract

Copper sulphate ingestion (accidental or deliberate) is a rare form of poisoning usually limited to the Indian subcontinent. Though the rates are on the decline, it is essential that physicians are aware of its lethal complications and management strategies. The main complications of copper sulphate ingestion include intravascular haemolysis, methaemoglobinaemia, acute kidney injury and rhabdomyolysis. The lethal dose can be as small as 10 grams. We have explored the complications of acute copper sulphate poisoning with examples from two case presentations. We also recommend measures for prevention of such events.

## Introduction

Copper sulphate is an easily accessible chemical in Sri Lanka that is sold over the counter. It is commonly used as a pesticide, in leather industry and also in making home-made glue. Burning of copper sulphate in houses and shops (as a good luck charm and for religious activities) is a common practice among Buddhists and Hindus. The marine blue colour of the hydrated form of copper sulphate crystals is attractive to children and is a reason for inadvertent poisoning. However, many admissions due to copper sulphate poisoning in adult medicine are acts of deliberate self harm. Ingestion of significant quantities of copper carries a risk of multi organ failure and death [1]. Unfortunately, many physicians are not familiar with the complications and management of this unusual form of poisoning that has become a rarity. We share our experience in treating two such patients and discuss the clinical manifestations, management issues and suggestions for prevention.

## Case presentation 1

A 26 year old male was admitted following ingestion of approximately 30 grams of copper sulphate 48 hours prior to admission. He had been managed initially at the local hospital for 2 days, with penicillamine (500 mg 6 hourly) and symptomatic treatment. On admission, he complained of epigastric and right hypochondrial pain, vomiting, haematemesis and melaena. There were no other bleeding manifestations. His urine output had reduced and was noted to be dark in colour. He did not complain of shortness of breath at rest or on exertion.

On examination, he was febrile, pale, icteric and cyanosed (chocolate cyanosis). Pulse rate was 80/min and blood pressure was 120/80 mmHg. Examination of the precordium and the respiratory system were unremarkable. Epigastric and right hypochondrial regions were tender with a firm and tender liver that was palpable 2 cm below costal margin. The epigastric pain was severe and radiated to back. Other organs were not palpable. He was conscious, rational and the neurological examination was normal.

During the hospital stay, he developed several complications of copper sulphate poisoning, namely; haemolysis, acute kidney injury, erosive gastritis with upper gastrointestinal bleeding, methaemoglobinaemia and hepatitis. The investigation results are summarized in table 1.

**Table 1:** Summary of investigation results of the first patient from admission up to day 30 (D30)

Investigation (Reference range)	D1	D4*	D15	D20	D30
Haemoglobin (g/l) (13-16)	14.9	10.4	9.5	8.9	11.6

Platelets (× 10^9^/l) (150-450)	337	268	138	254	276

White blood cells (× 10^9^/l) (4-11)	19	21.2	10.3	6.3	5.7

AST (U/L) (< 40)	138	-	52	62	28

ALT (U/L) (< 40)	27	-	10	10	10

ALP (U/L) (< 360)	-	-	406	242	225

Total Bilirubin (ɥmol/l) (18-21)	-	-	42	21	18

Protein (g/L) (60-85)	-	-	67	56	63

Albumin (g/L) (36-48)	-	-	37	28	34

Globulin (g/L) (23-35)	-	-	30	28	29

Blood urea (mg/dl) (< 40)	52	-	-	137	

Creatinine (ɥmol/l) (< 120)	131	349	716	756	453

Na+(meq/l) (136-148)	137	140	135	142	145

K+ (meq/l) (3.5-5.3)	4.2	6.9	3.1	4.5	4.4

Cl- (meq/l) (95-105)	109	-	-	-	

Fasting blood sugar (mg/dl) (65-110)	132	-	-	-	

Serum Amylase(U/l) (25-125)	-	743	3975	2380	460

*Haemodialysis was started at D4

Within the first four days since admission, he became anuric and oedematous. Pleural effusions and mild ascites were noted on ultrasonography which also showed evidence of acute renal parenchymal disease. The serum creatinine levels progressively increased with concurrent hyperkalaemia. He had to be supported with haemodialysis for nearly 4 weeks (started at day 4 since admission) until his renal functions recovered.

There was a significant drop in haemoglobin level which would have been contributed by the copper induced haemolysis (increased total bilirubin and indirect bilirubin fraction, evidence from blood picture, increased reticulocyte index) and upper gastrointestinal bleeding due to erosive gastropathy (confirmed later with upper GI endoscopy). He was transfused with 3 units of red cell concentrates to maintain the haemoglobin level above 11 g/l. An intravenous omeprazole infusion was continued for 4 days.

The methaemoglobinaemia could not be confirmed by biochemically as the assay was unavailable at the hospital. It was diagnosed clinically based on the presence of chocolate coloured cyanosis and a peripheral saturation of 85% (with pulse oximetry) when the central saturation (as measured by arterial blood gas) was 98%. The patient was treated with IV methylene blue 2 mg/kg (in 5% dextrose) for the methaemoglobinaemia. The cyanosis responded to the initial dose and further dosing was unnecessary. The hepatitis was managed symptomatically and the liver enzyme levels were not markedly altered.

His abdominal pain and clinical features resembled acute pancreatitis (epigastric pain radiating to back, lack of hard abdominal signs despite severe pain) and interestingly the serum amylase level was also very high. He was treated as for acute pancreatitis and the serum amylase level returned to baseline as the overall condition improved.

Chelation therapy was started with penicillamine at a dose of 500 mg 6 hourly. The patient made a good clinical recovery and was discharged on day 38 (D38) after admission. He had no further complaints and his serum creatinine level was within reference range in a follow up visit 2 weeks later.

## Case presentation 2

A 45 year old, previously healthy man was admitted two hours after ingestion of approximately 50 grams of highly concentrated copper sulphate, in a suicidal attempt. Immediately after ingestion, he had vomited bluish gastric contents and later complained of body weakness and abdominal pain.

On examination, he was conscious, rational, hemodynamically stable with a pulse rate of 80 beats per minute and a blood pressure of 120/80 mmHg. The respiratory, abdominal and nervous system examination was unremarkable.

His investigation results are summarized in table 2.

**Table 2:** Summary of investigation results of the third patient

Investigation	D0	D2-5	On discharge
Haemoglobin (g/l)	14.1	6.9	12.6

White blood cells (x 10^9^/l)	9.9	40.6 (85% neutrophils)	9.6

Serum Creatinine (μmol/l)	61	256 - 621	136

AST (U/l)	-	1077	76

ALT (U/l)	-	257	42

Total bilirubin (μmol/l)	-	265	32

Direct bilirubin (μmol/l) (< 4)	-	124	18

PT/INR	-	2.2	1.1

C-reactive protein (CRP) (< 5 mg/l)		7.3	5

Reticulocyte count (0.25-2.5%)		9%	

The patient developed severe epigastric pain with loose stools and melaena within 48 hours since admission. The haemoglobin dropped to 6.3 g/l with evidence of intravascular haemolysis. By day 4 he developed severe aspiration pneumonia and had to be electively intubated and ventilated. Haemodialysis was initiated from day 5 onwards due to concurrent acute kidney injury. Dialysis was continued on an every other day basis for 10 days till the renal functions started recovering.

The patient was managed with supportive care and chelation therapy (oral penicillamine 500 mg, 6 hourly). Erosive gastropathy was treated with an intravenous omeprazole infusion and low haemoglobin was corrected with red cell concentrate transfusions. Aspiration pneumonia was treated with intravenous meropenem 1 g 8 hourly as per antibiotic sensitivity pattern. He required mechanical ventilation for eight days in the intensive care unit and was subsequently transferred to the ward and observed for further complications. The patient made a gradual convalescence and was discharged after 25 days of hospital stay. He did not develop any further complications on follow up

## Discussion

Ingestion of more than 1 g of copper sulphate results in manifestation of symptoms of toxicity [2]. However, this is only a rough threshold for toxicity and depends on individual factors. Mortality in cases of severe poisoning is high and the lethal dose of ingested copper sulphate is between 10-20 g [3].

The clinical manifestations of copper sulphate poisoning include; erosive gastropathy, intravascular haemolysis, methaemoglobinaemia, hepatitis, acute kidney injury and rhabdomyolysis. Arrhythmias and seizures are also reported probably secondary to other organ system involvement [1,4].

Common gastrointestinal manifestations of copper poisoning are predominantly due to corrosive injury. Haematemesis and melaena are observed with severe overdose probably due to bleeding from mucosal injury. Liver gets damaged early in copper poisoning as the majority of absorbed copper is deposited in liver after being delivered from the portal circulation. Acute liver failure following tissue necrosis can occur due to direct copper toxicity [2,5].

Two major haematological manifestations of copper sulphate poisoning are intravascular haemolysis and methaemoglobinaemia [1]. Intravascular haemolysis can start as early as within the first 24 hours since ingestion and is due to the direct oxidative damage to erythrocyte membranes. The haemolysis can be rapid and severe with drastic drops in the haemoglobin level. The Cu^2+ ^ion oxidizes the Fe^2+ ^ion in haemoglobin to Fe^3+ ^resulting in its conversion to methaemoglobin [2]. This manifests as cyanosis and loss of oxygen carrying capacity of blood.

Acute kidney injury is a much commoner manifestation of toxicity with some case reports having an incidence as high as 40-60% [6-9]. The possible mechanisms of kidney damage include; pre-renal failure due to dehydration (vomiting, diarrhea, reduced fluid intake), haemoglobinuria, sepsis, rhabdomyolysis, direct copper toxicity on proximal tubules and secondary effects of multi organ dysfunction. The recovery of renal function following copper sulphate ingestion is observed to be slow and incomplete. In the first patient it took nearly 5 weeks before he was independent of dialysis.

The severe epigastric pain (unusual for gastropathy alone) and elevated amylase level in the first patient indicated the possibility of acute pancreatitis. It is not a recognized complication of copper sulphate poisoning. However, a rise in serum amylase cannot be attributed to pancreatitis alone (even acute kidney injury is associated with a rise in serum amylase) and we could not perform a CT scan with intravenous contrast in the acute phase of illness due to the renal impairment. Therefore, in this patient, pancreatitis could not be confirmed. This observation has also been reported by Gunay et al [10] previously in a single patient of a case series. We suggest that serum amylase level should be monitored carefully in patients with copper sulphate poisoning and the possibility of acute pancreatitis must be considered though it may be difficult to confirm it by imaging due to concurrent renal failure.

The management of copper sulphate poisoning centers on four key principles; a) reducing absorption b) close observation for complications c)supportive therapy and d) chelation therapy to remove active copper from the body.

After ingestion, the contact damage to mucosa can be minimized by drinking large quantities of milk and water [1]. Dilution reduces the direct mucosal injury. Emesis must be avoided as repeated exposure of the oesophagus to the corrosive agent may inflict further damage on the mucosa. Some authors recommend the use of activated charcoal (50 grams dissolved in 200 ml of water, administered in multiple doses if necessary at 6 hourly intervals) to reduce absorption, but it is of unproven benefit [1].

All complications mentioned above must be monitored for from the first 24 hours onwards (daily full blood counts, serum electrolytes, liver and renal function tests). If the patient had vomited, aspiration pneumonia or a chemical pneumonitis should be anticipated. It would be worthwhile to have a baseline chest roentgenogram in this regard. Evidence for haemolysis must be looked for with daily blood pictures and reticulocyte counts. If the patient is having spontaneous bleeding, the coagulation profile should be requested and if he is cyanosed, methaemoglobin levels should be assessed.

Anaemia from haemolysis or bleeding must be corrected with transfusion with red cell concentrates. Methaemoglobinaemia is treated with methylene blue (intravenous injection of 1-2 mg/kg/dose and repeated if cyanosis persists beyond one hour)[11]. However, high doses of methylene blue itself can cause haemolysis and it is contraindicated in G6PD deficiency. Alternatives to methylene blue in such situations would be hyperbaric oxygen and ascorbic acid (a much weaker reducing agent)[11].

Renal failure must be recognized early by careful monitoring of serum creatinine and urine output. Though dialysis is ineffective in clearing copper from the body, it will be an essential requirement to sustain life in event of acute kidney injury [1]. Peritoneal dialysis is ineffective in maintaining the relatively longer requirement for dialysis until the renal functions recover. However, it is an option when haemodialysis facilities are not available.

Chelation therapy in copper sulphate poisoning aims at removing ingested copper from the body. The efficacy of these chelating agents is unproven. Penicillamine is a commonly used chelating agent at a dose of 1-1.5 g/d in 2-4 divided doses [1]. Both our patients received a high dose of oral penicillamine without any ill effects. It must be used carefully in places without access to renal replacement therapy as penicillamine is nephrotoxic. Intramuscular administration of dimercaprol or British anti-Lewisite (BAL) (3-5 mg/kg/dose with four hourly administration in first two days and tailed off over a total of 7-11 days) is recommended when oral administration of penicillamine is difficult or contraindicated (severe corrosive injury in alimentary tract)[12]. Some authors question its efficacy when compared to penicillamine. Edetate Calcium disodium is another option and some recommend it as the first line therapy when the use of penicillamine is deemed unsafe [13]. There are case reports of patients with copper poisoning being successfully treated with edetate calcium disodium followed up by either oral penicillamine or dimercaprol [13,14]. However, edetate calcium disodium also carries the risk of acute tubular necrosis and have to be carefully administered with dose reductions in presence of acute kidney injury [13]. The duration of chelation therapy is not established by evidence. It is recommended to treat with chelators as long as the serum copper level remain above normal [13].

Despite being rare, intoxication with copper sulphate can be fatal. Therefore it is important to concentrate on prevention of copper sulphate ingestion by measures such as;

1. stopping over the counter sale of copper sulphate and restriction of purchase, distribution and sale to authorized agents only

2. alternately, over the counter availability of copper sulphate can be limited to large sized crystals; cases of poisoning are usually by dissolving the fine power like pulverized form of the compound

## Conclusions

Ingestion of copper sulphate is a rare form of poisoning. However, given the complications and high mortality rates, it's essential that clinicians are familiar with the management of such patients. The effects of poisoning can have deleterious effects on the upper digestive tract, kidneys, liver and blood (intravascular haemolysis, methaemoglobinaemia). We have observed that one of our patients had typical clinical features of acute pancreatitis (not a recognized complication) with an elevated serum amylase level. It may be worthwhile that physicians treating such patients assess the serum amylase level during the acute illness. We further recommend that restricting the availability of the pulverized powdered form of the compound in the open market might be an effective measure in preventing deliberate self harm by ingestion of copper sulphate.

## Consent

Written informed consent was obtained from the patients for publication of this case report. Copies of the written consent are available for review by the Editor-in-Chief of this journal.

## List of abbreviations

Abbreviations are explained where they are first used in text

## Competing interests

The authors declare that they have no competing interests.

## Authors' contributions

CR, CSSKG, AG have participated in designing, article search, information coding and writing of the manuscript. They were also responsible for the clinical care of the first and second patients. VP, SW and HF were responsible for the clinical care of the third patient. All authors have read and approved the final manuscript.

## Authors' information

CR, SW and CSSKG (MBBS) are registrars in Internal Medicine attached to the National Hospital of Sri Lanka. VP (MBBS) is Medical Officer of the medical intensive care unit of the National Hospital of Sri Lanka. HF (MBBS, MD, FRCP, FCCP) is Consultant Physician of Ward 49, National Hospital of Sri Lanka. AG (MBBS, MD, MPhil, FRCP) is Consultant Physician and Professor in Medicine attached to the Department of Clinical Medicine, Faculty of Medicine, University of Colombo, Sri Lanka.
